# Frequency of atrial thrombus formation in patients with atrial fibrillation under treatment with non-vitamin K oral anticoagulants in comparison to vitamin K antagonists: a systematic review and meta-analysis

**DOI:** 10.1186/s40001-018-0350-9

**Published:** 2018-10-23

**Authors:** Stefan Reers, Georg Karanatsios, Matthias Borowski, Michael Kellner, Michael Reppel, Johannes Waltenberger

**Affiliations:** 1 Department of Cardiovascular Medicine, University Hospital Münster, 48149 Münster, Germany; 2 Institute of Biostatistics and Clinical Research, University of Münster, Münster, Germany; 3 Neurologic Clinic, St. Vincenz-Krankenhaus, Paderborn, Germany; 4 Medical Practice for Cardiology and Angiology Landsberg, Landsberg am Lech, Germany

**Keywords:** Thrombus, Non-vitamin K antagonist oral anticoagulants, NOAC, Atrial fibrillation

## Abstract

**Background:**

To assess the frequency of left atrium/left atrial appendage (LA/LAA) thrombus under treatment with non-vitamin K oral anticoagulants (NOACs) in comparison with vitamin K antagonists (VKAs) in patients with non-valvular atrial fibrillation (AF).

**Methods:**

PubMed, Web of Science™, EMBASE and the Cochrane Library databases were searched for studies comparing NOACs with VKAs in AF patients who underwent diagnostic transoesophageal echocardiography (TEE).

**Results:**

A total of four trials were considered eligible and were included in the meta-analysis. Four RCTs comprising *n* = 2397 AF patients (NOACs: *n* = 1412, VKAs: *n* = 985) were included in the meta-analysis. The frequency of LA/LAA thrombus formation under treatment with NOACs was similar to VKAs [odds ratio (OR) 1.14, 95% confidence intervals (95% CIs) 0.97–1.65, *p* = 0.48]. Both treatment groups revealed an approximately 5% frequency of thrombus formation, although a precise calculation is not possible due to Simpson paradox. Indications of heterogeneity between the included trials were not found (*χ*^2^ test *p* = 0.99, *I*^2^ = 0%).

**Conclusions:**

The findings of this meta-analysis suggest that NOACs are similar to VKAs regarding the frequency of LA/LAA thrombus in patients with AF. An unknown number of patients in the original studies did not receive sufficient anticoagulation for at least 3 weeks prior to TEE examination, and therefore the present results should be interpreted with caution. Systematic review registration—http://www.crd.york.ac.uk/PROSPERO. Unique identifier: PROSPERO CRD42017059293.

**Electronic supplementary material:**

The online version of this article (10.1186/s40001-018-0350-9) contains supplementary material, which is available to authorized users.

## Background

Non-valvular atrial fibrillation (AF) is the most common cardiac arrhythmia and is associated with a considerable risk of stroke, systemic embolism (SE), heart failure and all-cause mortality [1]. Without oral anticoagulation, there is fivefold increased age-adjusted risk of AF-associated stroke [2]. For several decades, OAC with vitamin K antagonist (VKA) was the standard therapy in reducing risks of AF-associated stroke and SE, with a relative risk reduction of 62% [3]. The CHA_2_DS_2_-VASc score is the risk score most commonly used to determine the indication for anticoagulation. It represents a further development of the established CHADS_2_ score (congestive heart failure, hypertension, age ≥ 75 years, diabetes mellitus and prior stroke or transient ischaemic attack), and adds further stroke risk factors (vascular disease in the form of prior myocardial infarction, plaque in aorta and peripheral artery disease, age 65–74 years and female sex) [4]. According to the current American and European guidelines, patients with a CHA_2_DS_2_-VASc score ≥ 2 have an increased stroke risk necessitating anticoagulation therapy [5, 6]. Since VKA therapy has several limitations, such as inter-patient and intra-patient variability of drug dose, regular monitoring to ensure therapeutic anticoagulation within a target international normalised ratio (INR) range (2.0–3.0) is required [7]. Time in therapeutic range (TTR) ≥ 65%, to ensure adequate stroke risk prevention, is rarely achieved, even in large trials [8–11]. The drug compliance and TTR are less optimal in real life than in RCTs. Non-vitamin K antagonist oral anticoagulants (NOACs) have therefore been developed with direct inhibition of the coagulation cascade and without the need for routine coagulation monitoring. The NOAC group includes the direct thrombin inhibitor dabigatran and the direct factor Xa (FXa) inhibitors apixaban, edoxaban and rivaroxaban. In large clinical phase III trials, all four NOACs were effective as VKA in preventing stroke and SE with lower rates of haemorrhagic stroke [8–11]. A meta-analysis of all four NOACs demonstrates that the risk of stroke or SE was reduced by 19% compared to VKA (relative risk ratio 0.81; 95% confidence interval 0.73–0.91, *p* < 0.0001). The NOACs also show similar or lower rates of major or clinically relevant non-major bleeding events [12]. Consequently, all four NOACs were approved by the US Food and Drug Administration (FDA) in 2010 (dabigatran), 2011 (rivaroxaban), 2012 (apixaban) and 2015 (edoxaban) for patients with AF [13].

The frequency of left atrial (LA)/left atrial appendage (LAA) thrombus formation in patients with AF varies depending on anticoagulation (non vs. anticoagulation), type of treatment (concomitant treatment with acetylsalicylic acid vs. OAC alone), targeted INR values and TTR, type of AF (paroxysmal AF vs. non-paroxysmal AF), LAA morphology (chicken wing vs. non-chicken wing), LA size, increased left ventricular end-diastolic volume, ejection fraction (EF), inappropriate duration of anticoagulation < 3 weeks, metabolic syndrome, diabetes mellitus, CHADS_2_ and CHA_2_DS_2_-VASc score [14–24]. Retrospective studies revealed frequencies of LA/LAA thrombus in AF patients without anticoagulation therapy in the range of 13.0–19.0% [18, 24, 25]. The EMANATE trial, a randomised, active-controlled, open-labelled study showed a prevalence of thrombus formation in anticoagulation-naive AF patients of 7.1% [26]. The frequencies of thrombus formation under treatment with VKA vary between 3.5% and 17.8% [16, 17, 20, 21, 27]. Controlled therapeutic anticoagulation with VKA (INR 2.0–3.0) exhibited the lowest rates of intracardiac thrombus formation among retrospective studies to be of 0.6–7.7% [15, 19, 28].

The gold standard and most simple method for the exclusion of LA/LAA thrombus is TEE. In AF patients of more than 48-h duration, insufficient or no anticoagulation, therapeutic anticoagulation for at least 3 weeks prior to cardioversion or TEE is recommended. However, little is known about the frequency of LA/LAA thrombus under anticoagulation with NOACs in comparison to VKAs. The objective of this meta-analysis was thus to evaluate the frequency of LA/LAA thrombus formation in patients with AF under treatment with non-vitamin K oral anticoagulants in comparison to vitamin K antagonists.

## Methods

### Search strategy

In accordance with the Cochrane Handbook recommendations and Preferred Reporting Items for Systematic reviews and Meta-Analyses (PRISMA) guidelines [29, 30], we performed a systematic review of the literature and searched Pubmed, Web of Science™, EMBASE and the Cochrane library database using keywords, from the beginning of the database to 2 April 2017. The search strategy and the review protocol are available in the data supplement (Additional files 1, 2). We looked for eligible studies with a randomised controlled design and reported on thrombus formation under treatment with NOAC in comparison with VKA. We checked the reference lists of all suitable studies to identify additional trials that were not found in the primary search. The present systematic review and meta-analysis were undertaken without funding.

### Search management

Two different authors (SR and GK) performed searches in the aforementioned databases with the listed keywords, as described in the PRISMA guidelines. All potential studies were selected and checked in duplicate. In case of disagreement, consensus was achieved with a third author. On the basis of the title and abstract, obviously irrelevant articles were excluded. The remaining articles were examined on the basis of the inclusion criteria. Only phase II, III and/or IV randomised controlled trials (RCTs) investigating thrombus formation under treatment with one of the NOACs compared to VKA were selected. The primary outcome was the frequency of LA/LAA thrombus formation under treatment with NOAC and/or VKAs.

### Meta-analysis

The meta-analysis was performed following the instructions and recommendations of the Cochrane Handbook for Systematic Reviews of Interventions [31] and Cochrane Handbook for Systematic Reviews of Interventions Version 5.1.0 [29]. Due to low event rates, the odds ratios (ORs)^1^ and 95% confidence intervals (95% CIs) of the individual studies, as well as the pooled OR and its 95% CI, were estimated using the Petos method (fixed-effects model), and presented as forest plots. We also estimated study-individual and pooled risk ratios (RRs)^2^ and risk differences (RDs)^3^ with corresponding 95% CIs using the Mantel–Haenszel method, assuming a fixed-effects model. Heterogeneity was evaluated with a *χ*^2^ test and the *I*^2^ statistic and funnel plots^4^ were created to assess publication bias. All analyses were carried out using RevMan 5.3 (The Nordic Cochrane Centre, The Cochrane Collaboration, 2011). The protocol was published at the PROSPERO website (http://www.crd.york.ac.uk/PROSPERO/) with registration number CRD42017059293.

## Results

### Search results

**Fig. 1:**
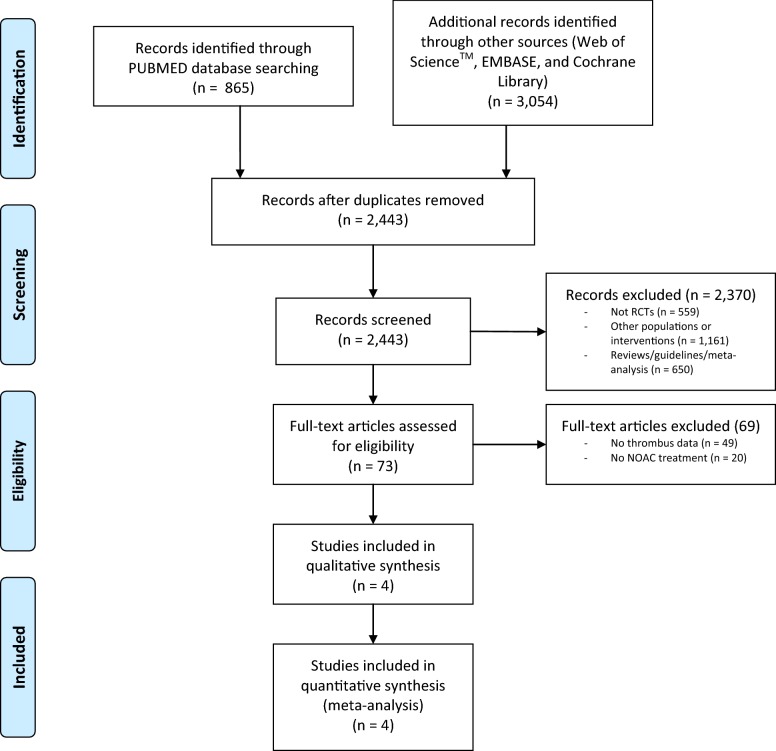
Search strategy according to the PRISMA guidelines

Our electronic database search identified a total of *n* = 3919 records (Fig. 1). After removal of duplicates, *n* = 2443 records were screened. Of the *n* = 2443 records, we identified *n* = 559 as non RCTs, *n* = 1161 as other populations or interventions and *n* = 650 as reviews/guidelines/meta-analysis. *N* = 73 full-text articles were assessed for eligibility. As there were no thrombus data (*n* = 49) and no data about NOAC treatment (*n* = 20), *n* = 69 articles were excluded. We identified four RCTs that fulfilled the inclusion criteria [32–35]. The baseline characteristics are listed in Table 1. All four trials investigated the outcome after cardioversion under treatment with NOACs or VKAs. TEE-guided cardioversions were performed only in a subgroup of AF patients, and results under anticoagulation treatment were recorded.

**Table 1:** Baseline characteristics of RCTs included in this review

Trial	Trial design	NOAC	Proportion of included patients	Sample size, n		Mean age (years)		Number of female (%)		Mean CHADS_2_ score		TTR	Number of TOE (%)		Ref.
				NOAC	VKA	NOAC	VKA	NOAC	VKA	NOAC	VKA		NOAC	VKA	
ARISTOLE subgroup	Post-hoc analysis of a double-blinded RCT	Apixaban	171 (31.7%)	265	275	67	67	72 (27%)	74 (27%)	1.8	1.9	59.0%	86 (32%)	85 (31%)	[32]
RE-LY subgroup	Post-hoc analysis of an open-label RCT	Dabigatran 110 mg BD	1183 (53.8%)	647	664	NR	NR	NR	NR	NR	NR	NR	168 (26%)	86 (13%)	[33]
		Dabigatran 150 mg BD		672		NR		NR		NR			161 (24%)		
ENSURE-AF	Open-label RCT to determine events after CV	Edoxaban	415 (20.9%)	1095	1104	64	64	374 (34%)	382 (35%)	2.6	2.6	70.8%	589 (54%)	594 (54%)	[31]
X-VeRT	Open-label RCT to determine events after CV	Rivaroxaban	628 (41.8%)	1002	502	65	65	275 (27%)	135 (27%)	NR*	NR*	NR	410 (41%)	218 (43%)	[34]

*ARISTOTLE* apixaban for the prevention of stroke in subjects with atrial fibrillation trial, *BD* twice daily, *CV* cardioversion, *ENSURE-AF* the edoxaban vs. warfarin in subjects undergoing cardioversion of atrial fibrillation study, *NOAC* non-vitamin K antagonist oral anticoagulants, *NR* not reported, NR* not reported as mean value, *RCT* randomised controlled trial, *Ref.* Reference, *RE-LY* randomised evaluation of long-term anticoagulation therapy trial, *TOE* transesophageal echocardiography, *TTR* time in therapeutic range, *X-VeRT* explore the efficacy and safety of once-daily oral rivaroxaban for the prevention of cardiovascular events in patients with non-valvular atrial fibrillation scheduled for cardioversion

### Risk of bias assessment

The Cochrane collaboration tool was used by two authors to determine risk of bias [29]. The risk of bias is divided into the following six domains: random sequence generation, allocation concealment, blinding of participants, personnel and outcome, incomplete outcome data, selective outcome reporting, other sources of bias. Potential sources of bias identified were the open-label design in two RCTs. The risk of bias assessment can be found in the supplemental data (Additional file 3).

### Meta-analysis

**Fig. 2:**
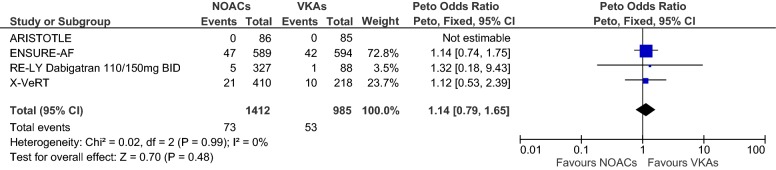
Forest plot to compare the frequencies of thrombus under treatment with NOACs vs. VKAs; odds ratios and 95% CIs were estimated using Petos method (fixed-effects model)

**Fig. 3:**
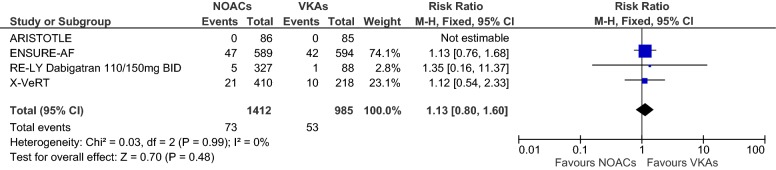
Forest plot to compare the frequencies of thrombus under treatment with NOACs vs. VKAs; risk ratios and 95% CIs were estimated using the Mantel–Haenszel method (fixed-effects model)

**Fig. 4:**
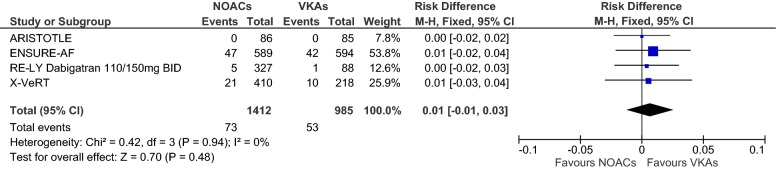
Forest plot to compare the frequencies of thrombus under treatment with NOACs vs. VKAs; risk differences and 95% CIs were estimated using the Mantel–Haenszel method (fixed-effects model)

Overall, we evaluated data from *n* = 2397 (*n* = 1412 received NOACs and *n* = 985 VKAs) patients in four RCTs. Figures 2, 3, 4 show forest plots presenting individual study and pooled ORs, RRs and RDs with 95% CIs. Since the ARISTOTLE trial reported no events in either arm, estimation of the individual study OR and RR was not possible here, but the RD could be estimated (see the meta-analysis section for an explanation). The ARISTOTLE was therefore not included in the estimation of the pooled OR and RR, but it was included in the estimation of the pooled RD. In each trial, the event percentage in the NOAC group was not greater than the event percentage in the VKA group (ARISTOTLE: 0/86 = 0% vs. 0/85 = 0%; ENSURE-AF: 47/589 = 8.0% vs. 42/594 = 7.1%; RE-LY: 5/327 = 1.5% vs. 1/88 = 1.1%; X-VeRT: 21/410 = 5.1% vs. 10/218 = 4.6%). The study-individual ORs were thus 1.14, 1.32 and 1.12 in favour of VKAs (Fig. 2). However, none of these ORs was “significant” since each 95% CI covered the one. The pooled OR estimate was 1.14 with 95% CI 0.79–1.65, and the test for an overall effect delivered a *p* value of *p* = 0.48: a considerable difference between NOACs and VKAs regarding the odds of LA/LAA thrombus formations could not be found. The results regarding the RRs (Fig. 3) were very similar, with a pooled RR of 1.13 (95% CI 0.80–1.60). The study-individual RDs (Fig. 4) were between 0% and 1%, and none of the 95% CIs suggested a difference between NOACs and VKAs. The estimated pooled RD (including the ARISTOTLE trial) was 1% with 95% CI − 1 to 3%.

**Fig. 5:**
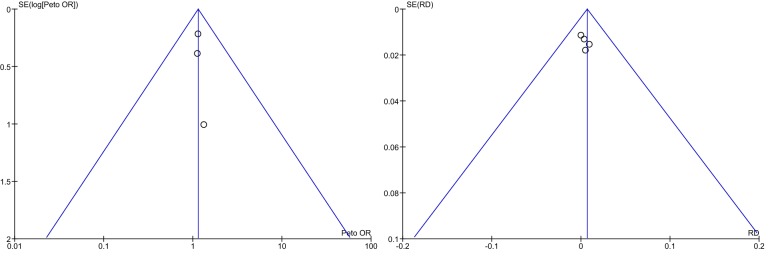
Funnel plots showing Peto ORs (left) and RDs (right); the left plot shows only three values since one RCT had no events in both arms, so that Peto ORs could not be estimated; the funnel plot of the RRs was very similar to that of the Peto ORs, and is therefore not shown here

Indications of heterogeneity between the studies could be found neither by the *χ*^2^ test (*p* = 0.99) nor by the *I*^2^ statistic (*I*^2^ = 0%), and the funnel plot (Fig. 5) did not suggest a high risk of publication bias. However, the small number of studies made a reliable assessment of heterogeneity and publication virtually impossible.

Finally, we note that the so-called Simpson paradox occurs if the absolute numbers of patients and events in the four trials are used for naïve calculations of pooled risks under NOACs and VKAs. If we calculated Risk_naive_ (NOACs) = (0 + 47 + 5+21)/(86 + 589 + 327 + 410), we would obtain Risk_naive_ (NOACs) = 5.2%. The same naïve calculation approach would yield RR_naive_ (VKAs) = 5.4%. With these naïve calculations, we observe that the pooled Risk is lower for NOACs, although NOACs had a higher risk in each study. In any case, the calculations of (pooled) odds ratios, risk ratios and risk differences that we used make Simpson paradox impossible.

## Discussion

In the present study, we investigated LA/LAA thrombus formation in AF patients under treatment with NOACs and VKAs, respectively. The results of this meta-analysis showed a similar incidence of thrombus formation (OR 1.14, 95%, CI 0.79–1.65, *p* = 0.48). Neither the *χ*^2^ test (*p* = 0.99) nor *I*^2^ = 0% revealed evidence of heterogeneity between the trials included in the analysis.

The intensity and type of anticoagulation have a considerable impact on LA/LAA thrombus formation in AF patients [18, 20]. The four trials revealed a frequency of thrombus formation in AF patients under treatment with NOACs and VKAs of about 5.0%. A precise calculation is difficult due to pronounced Simpson paradox. Nevertheless, the results of this meta-analysis are in accordance with the retrospective studies investigating the frequency of LA/LAA thrombus in AF patients with varying INR/TTR (3.5–17.8%) and controlled therapeutic anticoagulation (0.6–7.7%) [15, 16, 18–21, 27, 28]. It is notable that the approximately 5% rate of LA/LAA thrombus formation is considerably higher than the average stroke rate of < 1%. It’s reasonable to assume that not every thrombus detaches itself during cardioversion and not every stroke is clinically diagnosed.

The clinical standard for evaluating LA/LAA in AF patients is TEE. The current guidelines recommend at least 3 weeks of effective anticoagulation or TEE before cardioversion to exclude LA/LAA thrombus in patients with AF more than 48 h or unknown duration [13]. Sufficient therapeutic anticoagulation reduced the peri-procedural stroke and SE risk from 3.4% to < 1% [36, 37]. A current meta-analysis also revealed a peri-procedural stroke rate and SE risk rate of 0.41% and 0.61% in patients treated with NOACs and VKAs, respectively [38]. NOACs therefore seem to be a safe and effective alternative to VKAs in AF patients undergoing cardioversion.

The half-lives of NOACs range from 5 to 17 h, and the plasma levels are detectable up to 24 h after ingestion [39]. After 24 h, NOACs have little effectiveness [13]. An assessment of compliance with NOACs in patients undergoing cardioversion may thus be problematic in clinical routine. Due to the absence of regular monitoring, clinicians must rely on the patient’s valid statement. The routine use of TEE prior to cardioversion is therefore discussed intensively. On the other hand, in large RCTs, a drug intake of more than 80% is considered sufficient treatment and has demonstrated remarkable results [35]. The LAA is the most frequent origin of thrombus formation in AF patients, and patients with documented LA/LAA thrombus had a stroke or SE rate of more than 10% per year despite VKA treatment [40]. The established therapy for LA/LAA thrombus was low molecular heparin bridged with VKA treatment [5]. However, approximately 40% of intracardiac thrombus persist under VKA treatment [41]. The results of the X-TRA trial showed that resolved or reduced thrombus was evident only in 60.4% of patients under treatment with rivaroxaban. The EMANATE trial showed that in AF patients with LA/LAA thrombus, the rate of resolved thrombus was 52% under treatment with apixaban and 58% under therapy with heparin/VKA [26]. Data from the retrospective CLOT-AF registry revealed complete thrombus resolution in 62.5% AF patients [42]. Due to the different natures and heterogeneous study population of these trials, a direct comparison cannot be made, but rivaroxaban and apixaban seems to be an equivalent therapy for LA/LAA thrombus in AF patients. A further prospective trial evaluating the efficacy of dabigatran (RE-LATED AF-AFNET 7, REsolution of Left Atrial-appendage Thrombus-Effects of Dabigatran in patients with AF) is ongoing [43].

Our study has some limitations. First, the four trials included differed with respect to protocol, inclusion and exclusion criteria, study population, the CHADS_2_/CHA_2_DS_2_-VASc scores and a missing definition of LA/LAA thrombus. Second, the TTR is only available in the ARISTOTLE subgroup and the ENSURE-AF trial. Even in these studies the TTR ranges from 59.0 to 70.8%. The other two studies did not mention TTR data and may cause bias. Third, the determined heterogeneity (like the *χ*^2^ test and the *I*^2^) of the four studies must be interpreted with caution. According to the Cochrane handbook, a minimum of ten studies is recommended for using this method [29]. Similar to other high quality meta-analyses, only well-conducted trials have been included. Fourth, the weight of included studies diverges considerably. The majority involve the ENSURE-AF study, followed by the X-VeRT study. Data from the RE-LY study is in a minority and bias due an imbalance of more patients in each dabigatran group than in the warfarin group cannot be excluded. Data from the ASTISTOTLE trial was also not included in the calculation of OR and RR, because there were no events in either study arm. Fifth, the duration of anticoagulant treatment prior to TEE in most included trials is unknown and ranges from a few days (edoxaban) to 60 days (dabigatran) after randomisation. Sixth, an unknown proportion of patients in the original studies did not receive sufficient anticoagulation for at least 3 weeks, and therefore, the present results must be interpreted with caution.

## Conclusion

This meta-analysis evaluated the incidence of LA/LAA thrombus under treatment with NOACs in comparison to VKAs. The frequencies of LA/LAA thrombus in both treatment groups were similar (OR 1.14, 95% CI 0.97–1.65, *p* = 0.48). There were no observed indications of heterogeneity between the trials included (*χ*^2^ test *p* = 0.99, *I*^2^ = 0%). The frequency of thrombus formation under NOACs and VKAs was about 5%, although an exact calculation is not possible due to Simpson paradox.

### Clinical implications

The results of the present study show an LA/LAA thrombus formation of about 5%, despite sufficient anticoagulation. Imaging should be used to exclude thrombus formation before interventions and surgery of the LA/LAA in AF patients. Prior to cardioversion, clinicians must rely on the patient’s valid statement regarding continuous intake of NOACs. In cases of doubt, imaging should be performed to exclude intracardiac thrombus.

## Additional files

**Additional file 1.** Review protocol.

**Additional file 2.** Search strategy.

**Additional file 3.** Risk of bias summary: review authors' judgements about each risk of bias item for each included study. + indicates low risk of bias, − indicates high risk of bias, and no specification indicates unclear or unknown risk of bias.

## Footnotes

1 The odds ratio (OR) of group A vs. B is the ratio of two odds, i.e. OR (A vs. B) = odds (A)/odds (B), where odds (A) and odds (B) are the observed odds of LA/LAA thrombus formations in groups A and B, respectively.

2 The risk ratio (RR) of group A vs. B is the ratio of two risk estimates, i.e. RR (A vs. B) = risk (A)/risk (B), where risk (A) and risk (B) are the percentages of patients who had LA/LAA thrombus formation in group A and B, respectively. If risk (A) and risk (B) are zero, the RR cannot be computed. In this case, the risk difference (RD) is generally considered.

3 The risk difference (RD) of group A vs. B is the difference of two risk estimates, i.e. RD (A vs. B) = risk (A) − risk (B), where risk (A) and risk (B) are the percentages of patients who had LA/LAA thrombus formation in groups A and B, respectively.

4 A funnel plot is a common graphic used to assess whether a publication bias is given. Each dot in the plot represents a study. The *y*-axis shows the study precision (i.e. the standard error) and the *x*-axis shows the study's effect). A publication bias is unlikely if the funnel shape is symmetric. An asymmetric shape, however, is an indication for a publication bias.
