# Supplementary material for: Frequency of atrial thrombus formation in patients with atrial fibrillation under treatment with non-vitamin K oral anticoagulants in comparison to vitamin K antagonists: a systematic review and meta-analysis
Source: Eur J Med Res. 2018 Oct 23;23:49. doi: 10.1186/s40001-018-0350-9 (PMC6198509; doi:10.1186/s40001-018-0350-9)
Supplement: Supplementary file 1 — Additional file 1. Review protocol. [file 40001_2018_350_MOESM1_ESM.docx]

**Review Protocol**

| \| Title of the review \| \| --- \| | Frequency of atrial thrombus formation in patients with atrial fibrillation under treatment with non-vitamin K oral anticoagulants in comparison to vitamin K antagonists: a systematic review and meta-analysis | |
| --- | --- | --- | --- |
| \| First reviewer \| \| --- \| | Stefan Reers | |
| \| Team of reviewers \| \| --- \| | Georg Karanatsios, Michael Kellner, Michael Reppel | |
| \| Supervisor/Project PI \| \| --- \| | Michael Reppel & Johannes Waltenberger | |
| \| Clinical Portfolio Group \| \| --- \| | Stefan Reers & Georg Karanatsios, consultant physician, Department of Cardiovascular Medicine, University Hospital Münster; Michael Borowski, Institute of Biostatistics and Clinical Research, University of Münster; Michael Kellner, consultant physician, Neurologic Clinic, St. Vincenz-Krankenhaus, Paderborn; Michael Reppel, Department of Cardiology/Angiology/Intensive Care Medicine, University Heart Center Lübeck; Johannes Waltenberger, Department of Cardiovascular Medicine, University Hospital Münster | |
| \| Project title (if different from review title) \| \| --- \| | Frequency of atrial thrombus formation under anticoagulation | |
|  | | |
| \| Support– please state if advice/training or personnel required at each stage \| \| --- \| | | |
| \| SR overview \| \| --- \| | Johannes Waltenberger | |
| \| Protocol development \| \| --- \| | Stefan Reers & Georg Karanatsios & Michael Kellner | |
| \| Literature searching \| \| --- \| | Already had training from library on literature searching | |
| \| Quality appraisal \| \| --- \| | Advice from reading around the area | |
| \| Data Extraction \| \| --- \| | Stefan Reers & Georg Karanatsios | |
| \| Synthesis \| \| --- \| | Stefan Reers & Georg Karanatsios | |
| Writing up | Stefan Reers & Georg Karanatsios | |
|  | | |
| **1. Background to review**  Brief introduction to the subject of the review, including rationale for undertaking the review and overall aim | | |
| Non-valvular atrial fibrillation (AF) is the most common heart rhythm disorder and is associated with an increased risk of thrombogenic complications such as stroke and systemic embolism (SE) for which embolism of atrial or left atrium/left atrial appendage (LA/LAA) thrombus is the predominant underlying mechanism. Over decades the use of vitamin K antagonists (VKAs) was standard therapy for stroke/SE prevention in patients with AF. Due to development of non-vitamin K antagonist oral anticoagulants (NOACs; apixaban, dabigatran, edoxaban, and rivaroxaban) disadvantages of VKA therapy such as need of frequent INR control have been overcome and major bleeding risk has been shown to be reduced. According to the current American and European guidelines, in AF patients of >48h duration, insufficient or no anticoagulation, therapeutic anticoagulation for at least 3 weeks prior to cardioversion or transesophageal echocardiography (TEE) is recommended. However, the incidence of LA/LAA thrombus of the NOACs is unknown. Hence, objective of this meta-analysis was to evaluate the effect of NOACs *versus* VKAs on LA/LAA thrombus formation. | | |
|  | | |
| **2. Specific objectives** | | |
| To evaluate the evidence base of available randomised controlled trials (RCTs) comparing NOACs with VKAs for the frequency of LA/LAA thrombus formation, PubMed, Web of Science^TM^, EMBASE, and the Cochrane Library databases were searched in AF patients. | | |
|  | | |
| **3. a) Criteria for including studies in the review**  If the PICOS format does not fit the research question of interest, please split up the question into separate concepts and put one under each heading | | |
| i. Population, or participants and conditions of interest | Gender | male and female |
|  | Age | ≥18 years |
|  | Type of AF | AF of more than 48 hours |
| ii. Interventions or exposures | Thrombus formation under oral anticoagulation | |
| iii. Comparisons or control groups | NOACs vs. VKAs | |
| iv. Outcomes of interest | Incidence of thrombus formation under treatment of NOACs vs. VKAs | |
| v. Setting | Hospital admissions and/or secondary care | |
| vi. Study designs | RCTs | |
|  | | |
| **3. b) Criteria for excluding studies not covered in inclusion criteria**  Any specific populations excluded, date range, language, whether abstracts or full text available, etc. | | |
| Main items:  AF or atrial flutter due to reversible causes; history of heart valve disorders; increased bleeding risk and/or contraindication to oral anticoagulation and/or simultaneous treatment with both aspirin and a thienopyridine | | |
|  | | |
| **4. Search methods** | | |
| Electronic databases  Please list all databases that are to be searched and include the interface (eg NHS, EBSCO, etc) and date ranges searched for each | PubMed/MEDLINE, Web of Science^TM^, EMBASE, and the Cochrane Library | |
| Other methods used for identifying relevant research  ie contacting experts and reference checking | Reference checking and hand searching of these. | |
| Journals hand searched  If any are to be hand searched, please list which journals and date searched from, including a rationale. | No additional journals were hand searched | |
|  | | |
| **5. Methods of review** | | |
| Details of methods  Number of reviewers, how agreements to be reached and disagreements dealt with, etc. | Two main reviewers (SR and GK) and a third reviewer(MR) in cases of disagreements | |
| Quality assessment  Tools or checklists used with references or URLs | The search strategy is available in the data supplement. | |
| Data extraction  What information is to be collected on each included study. If databases or forms on Word or Excel are used and how this is recorded and by how many reviewers | Selected studies have been stored in word-, excel- or pdf-files.  Procedure: first, reviewer number 1 (SR) will check the records, followed by reviewer number 2 (GK), which will be done independently. In cases of disagreements between both initial reviewer, reviewer number 3 (MR) will also check the disparities. | |
| Narrative synthesis  Details of what and how synthesis will be done | Narrative synthesis will be done in accordance with the Cochrane Handbook recommendations and Preferred Reporting Items for Systematic reviews and Meta-Analyses (PRISMA) guidelines | |
| Meta-analysis  Details of what and how analysis and testing will be done. If no meta-analysis is to be conducted, please give reason. | The meta-analysis follows the recommendations of the Cochrane Handbook for Systematic Reviews of Interventions and Cochrane Handbook for Systematic Reviews of Interventions Version 5.1.0. Because events seems to be rare, Odds Ratios (ORs) and 95% confidence intervals (95 % CIs) of the individual studies as well as the pooled OR and its 95% CI will be estimate using Petos method (fixed-effects model). | |
| Grading evidence  System used, if any, such as GRADE | GRADE-score: 4 points; evidence quality: high | |
|  |  | |
| **6. Presentation of results** | | |
| Additional material  Summary tables, flowcharts, etc, to be included in the final paper | 1) Main characteristics of RCTs  2) Protocol  3) Search strategy according to the PRISMA guidelines  4) Search strategy  5) Risk of bias assessments  6) Forest plots and funnel plots | |
| Outputs from review  Papers and target journals, conference presentations, reports, etc | Publication in a high quality cardiovascular journal | |
|  |  | |
| **7. Timeline for review – when do you aim to complete each stage of the review** | | |
| Protocol | 2 weeks | |
| Literature searching | 4 weeks | |
| Quality appraisal | 1 weeks | |
| Data extraction | 1 weeks | |
| Synthesis | 1 weeks | |
| Writing up | 4 weeks | |
