# Supplementary material for: Frequency of atrial thrombus formation in patients with atrial fibrillation under treatment with non-vitamin K oral anticoagulants in comparison to vitamin K antagonists: a systematic review and meta-analysis
Source: Eur J Med Res. 2018 Oct 23;23:49. doi: 10.1186/s40001-018-0350-9 (PMC6198509; doi:10.1186/s40001-018-0350-9)
Supplement: Supplementary file 2 — Additional file 2. Search strategy. [file 40001_2018_350_MOESM2_ESM.docx]

**Search strategy**

#1. Atrial fibrillation. af

#2. Atrial fibrillations. af

#3. #1 OR #2

#4. Apixaban. af

#5. BMS 562247. af

#6. Eliquis. af

#7. Dabigatran. af

#8. BIBR 1048. af

#9. Pradaxa. af

#10. Dabigatranetexilat. af

#11. Edoxaban. af

#12. DU 176b. af

#13. Lixiana. af

#14. Savaysa. af

#15. Rivaroxaban. af

#16. BAY 597939. af

#17. Xarelto. af

#18. Betrixaban. af

#19. PRT054021. af

#20. Darexaban. af

#21. YM150. af

#22. Letaxaban. af

#23. TAK-442. af

#24. Eribaxaban. af

#25. PD 0348292. af

#26. Ximelagatran. af

#27. H 376/95. af

#28. Exanta. af

#29. NOAC. af

#30. NOACs. af

#31. DOAC. af

#32. DOACs. af

#33. Novel oral anticoagulant. af

#34. New oral anticoagulant. af

#35. Novel anticoagulant. af

#36. New anticoagulant. af

#37. #4. OR #5. OR #6. OR #7. OR #8. OR #9. OR #10. #11. OR #12. OR #13. OR #14. OR #15. OR #16. OR #17. OR #18. OR #19. OR #20. OR #21. OR #22. OR #23. OR #24. OR #25. OR #26. OR #27. OR #28. OR #29. OR #30. OR #31. OR #32. OR #33. OR #34. OR #35. OR #36.

#38. Randomized controlled trial.pt

#39. Randomized clinical trial.pt

#40. Controlled clinical trial.pt

#41. Prospective trial.pt

#42. Randomized trial.pt

#43. Clinical trial, phase II.pt

#44. Clinical trial, phase III.pt

#45. Clinical trial, phase IV.pt

#46. Controlled clinical study.pt

#47. #38. OR #39. OR #40. OR #41. OR #42. OR #43. OR #44. OR #45. OR #46.

#48. Animal.af

#49. Animals.af

#50. Case reports.af

#51. Letter.af

#52. #48. OR #49. OR #50. OR #51.

#53. #3. AND #37. AND #47. NOT #52.
