# Supplementary material for: Frequency of atrial thrombus formation in patients with atrial fibrillation under treatment with non-vitamin K oral anticoagulants in comparison to vitamin K antagonists: a systematic review and meta-analysis
Source: Eur J Med Res. 2018 Oct 23;23:49. doi: 10.1186/s40001-018-0350-9 (PMC6198509; doi:10.1186/s40001-018-0350-9)
Supplement: Supplementary file 3 — Additional file 3. Risk of bias summary: review authors' judgements about each risk of bias item for each included study. + indicates low risk of bias, − indicates high risk of bias, and no specification indicates unclear or unknown risk of bias. [file 40001_2018_350_MOESM3_ESM.docx]

Risk of bias summary: review authors' judgements about each risk of bias item for each included study. For interpretation: + indicates low risk of bias, - indicates high risk of bias, and no specification indicates unclear or unknown risk of bias.
